# Toponomics method for the automated quantification of membrane protein translocation

**DOI:** 10.1186/1471-2105-12-370

**Published:** 2011-09-19

**Authors:** Olga Domanova, Stefan Borbe, Stefanie Mühlfeld, Martin Becker, Ralf Kubitz, Dieter Häussinger, Thomas Berlage

**Affiliations:** 1 Fraunhofer Institute for Applied Information Technology FIT, Schloss Birlinghoven, Sankt Augustin, Germany; 2 RWTH Aachen University, Information Systems Group (Informatik 5), Ahornstraße 55, Aachen, Germany; 3 Bonn-Aachen International Center for Information Technology B-IT, Dahlmannstraße 2, Bonn, Germany; 4 Heinrich-Heine University of Düsseldorf, Medical Faculty, Department of Gastroenterology, Hepatology and Infectiology, Moorenstr. 5, Düsseldorf, Germany

## Abstract

**Background:**

Intra-cellular and inter-cellular protein translocation can be observed by microscopic imaging of tissue sections prepared immunohistochemically. A manual densitometric analysis is time-consuming, subjective and error-prone. An automated quantification is faster, more reproducible, and should yield results comparable to manual evaluation. The automated method presented here was developed on rat liver tissue sections to study the translocation of bile salt transport proteins in hepatocytes. For validation, the cholestatic liver state was compared to the normal biological state.

**Results:**

An automated quantification method was developed to analyze the translocation of membrane proteins and evaluated in comparison to an established manual method. Firstly, regions of interest (membrane fragments) are identified in confocal microscopy images. Further, densitometric intensity profiles are extracted orthogonally to membrane fragments, following the direction from the plasma membrane to cytoplasm. Finally, several different quantitative descriptors were derived from the densitometric profiles and were compared regarding their statistical significance with respect to the transport protein distribution. Stable performance, robustness and reproducibility were tested using several independent experimental datasets. A fully automated workflow for the information extraction and statistical evaluation has been developed and produces robust results.

**Conclusions:**

New descriptors for the intensity distribution profiles were found to be more discriminative, i.e. more significant, than those used in previous research publications for the translocation quantification. The slow manual calculation can be substituted by the fast and unbiased automated method.

## Background

Densitometric analysis provides information about the distribution of the objects of interest. If different biological states of a sample are analyzed, a quantitative comparison of the protein distributions can be performed. The current manual method is subjective and error-prone. An automated analysis can collect more data points and be more objective in the choice of locations measured.

### Biological model

We used canalicular bile salt secretion in liver tissue as a model to develop a workflow for automated microscopy image analysis. The canalicular membranes of adjacent hepatocytes, the most abundant liver cell population [1,2], which limit tiny biliary ducts (the canaliculi) [2], are of a particular interest here. Hepatocytes continuously secret bile acids across their canalicular membrane [3]. Cholestatic liver diseases can result from a dysregulation of transport proteins in the sinusoidal [4] and the canalicular membranes [5]. In rat liver, the multidrug resistance protein 2 (Mrp2) as well as the bile salt export pump (Bsep) are regulated on a short-term scale by retrieval from and insertion into the canalicular membrane in response to e.g. anisoosmolarity [6-13]. In induced experimental cholestasis, the amount of Mrp2 in the canalicular membrane is reduced compared to liver tissue from untreated rats. Internalized Mrp2 was found in intracellular vesicles [7,9]. Liver perfusion in rats demonstrated that hyperosmolarity leads to rapid retrieval of Bsep from the canalicular membrane, reduces bile acid secretion and results in cholestasis. These protein translocations can be detected by analyzing the corresponding fluorescent microscopy images and can be quantified by toponomics methods.

### Manual analysis

**Figure 1:**
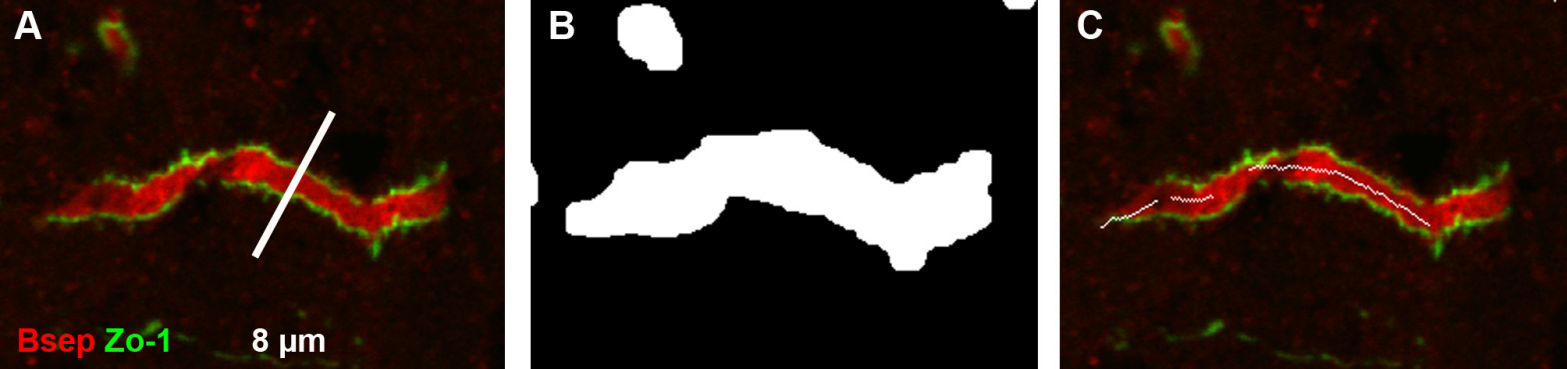
**Structure identification**. A. Representative canaliculus suitable for intensity profile extraction. It is straight and uniform, tight junctions are symmetrical, undisrupted and run in parallel. Protein intensity profiles of a length 8 *μ*m are extracted by measuring the pixel intensities of two channels (Bsep, red, functional marker protein; Zo-1, green, structural marker protein) perpendicular to the canaliculus. For example, one of the possibly extracted profiles is shown by a white line. B. Canalicular regions are extracted using the foreground-background detection function of the Zeta software [21]. Foreground mask of the canaliculus shown in A. White areas are considered for further processing. C. Skeletonization results.

Several toponomic localization studies of Bsep and Mrp2 were published so far [6,7,10,11,13,14] comparing transport protein distribution by manual processing of microscopic images. By means of immunofluorescence, proteins of interest (such as Bsep or Mrp2 and Zo-1, zonula occludens 1, a tight junction associated protein) were labeled with fluorescent markers. Zo-1 was used to localize the canaliculi, as the tight junctions delimitate the lateral from the canalicular membrane domains and the canalicular membrane of adjacent hepatocytes from the canalicular lumen. Two roughly parallel Zo-1 lines signal the presence of a canaliculus running parallel to the image plane of the microscope. An example of a suitable canaliculus is shown in Figure 1A. Such confocal microscopic images were manually assessed and processed previously. A standard dataset included 10 images of different tissue regions per biological condition, while only 10 measurements were performed on each image. We have developed a new method based on automated image analysis that substitutes the manual evaluation. This method is fast, unbiased and extracts information from over a thousand data points per image.

### Toponomics

The toponome is an account of the temporal and spatial organization of biological molecules, in particular proteins, within the structures of the organism, mainly on the level of cellular, subcellular and supercellular structures [15]. This analysis is also called topological proteomics or location proteomics [16,17].

Topological proteomics emphasizes the measurement of the spatial distribution of single or multiple proteins, while the term *toponome *describes the combined topological information in a cell and focuses on the underlying laws of this spatial arrangement. Laws in this context do not necessarily mean causal relations, but models of spatial distribution. The goal of toponomic modeling is to reduce image data to a compressed description of the spatial and structural relationships. The molecular markers can be classified as functional or structural. The structural marker defines reference structures while the functional marker is the object of interest in terms of spatial relations.

Spatial relations can be captured in different dimensionalities. Translocation studies are based on the quantitative assessment of marker concentrations in bounded regions (for example, nucleus-to-cytoplasm, see [18]). Protein co-localization studies can be based on measuring and evaluating isotropic distributions of distances between pixels [19]. If the former is described as a 2-dimensional analysis (regions), the latter should be regarded as 0-dimensional (points). The method described here is based on 1-dimensional modeling (orthogonal section to a membrane segment). A comparable method for tissue samples is described in [20].

## Results

Spatial relationships can be described as geometrical relations between functional objects (of different kinds) and between functional and structural entities. To derive spatial relationships in toponomic modeling, a multi-step workflow is required:

1. Establish structural entities by pattern recognition.

2. Establish a representative group of objects, which we can define geometry relations for.

3. Collect the geometric relations (such as distances) of the population of interest as a density plot into a histogram.

4. Evaluate the shape of the histogram with respect to the expectations from a biological model. Behaviors distinguished in the biological model cause different distributions (histogram shapes). Ideally, a single numerical descriptor is derived that discriminates behaviors.

While the calculations (3) and (4) can easily be performed automatically, (1) and (2) are more challenging, in particular in tissue sections. (1) and (2) are highly relevant, though, for the robustness of the overall method. To capture changes in the spatial distribution with high sensitivity, establishing a suitable numerical descriptor is also very important.

### Algorithm

**Figure 2:**
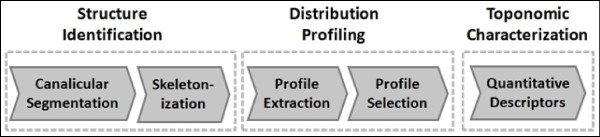
**Schematic representation of the developed automated workflow for the protein translocation analysis**. Canaliculi are identified in the images by foreground-background detection, followed by cleaning from noise using morphological operations and thresholding. Foreground regions are then skeletonized and pruned. Further, fluorescence intensity profiles are extracted perpendicular to the selected skeleton fragments. Profiles undergo the selection by empirically identified criteria and ranking according to the quality parameters. Zones are identified in average intensity profiles and numerical descriptors are evaluated. Wilcoxon rank sum test is applied to compare datasets.

We have specified and implemented a workflow for the automated quantification of the protein translocation consisting of several methods. Figure 2 illustrates the complete algorithm. The individual steps and the reasoning behind their choice is presented in the following sections.

#### Structure identification

As the first step, canalicular membranes are identified in the fluorescent microscopy images using the foreground-background detection function of the software Zeta [21], which implements a supervised learning approach to image region detection [22-24]. Representative examples of foreground and background regions (e.g. canalicular membranes and cellular lumen, respectively) are manually labeled in the image as training data for the machine learning. Within a square window a number of texture features are calculated [25-27]. The machine learning algorithm builds a classification model and extracts bit masks for the background and foreground of the whole image. High intensity fragments with high contrast to surrounding pixels are discriminated from low contrast background [28]. If a dataset is homogeneous and all microscopic images have a similar intensity range, the foreground detection can be trained on one image and applied to the whole dataset. In case of inhomogeneous data, training examples from several images have to be used. Figure 1B shows the foreground detection result of Figure 1A. The foreground regions (white) are considered for further processing.

In the next step, the foreground regions are refined and cleaned from noise. Morphological opening [29] deletes tiny objects, which might have been created by the foreground-background detection. Then, all small objects are deleted from the foreground mask to exclude potentially damaged or incomplete canaliculi. Subsequent morphological closing eliminates gaps, which were possibly introduced by morphological opening. A final cleaning step eliminates left over small objects.

In the following step, skeletonization of the foreground regions is performed according to Euler characteristics [30]. The obtained skeleton is pruned by deleting short branches that are attached to the main skeleton line. Pixels of the shorter branches are deleted one by one from four directions until no further deletion is possible. Only those parts of the skeleton are left that extend in the direction of the long axis of the membrane segments.

Pruning the skeleton is motivated by the manual strategy of selecting membrane segments for the intensity profile extraction. Only long, clean and unbranched membrane segments are suitable for analysis. Therefore, smaller foreground fragments and branching points with their neighbors are deleted from the skeleton (see Figure 1C).

#### Marker distribution profiling

Intensity distribution profiles are retrieved for the proteins of interest (Bsep, Zo-1) orthogonally to the skeleton. At every pixel of the skeleton the following operations are performed. Firstly, a tangent is fitted to the skeleton line at this pixel, so that the direction of this particular membrane segment is identified. Then, an orthogonal line is drawn through this pixel that spreads equally to both sides of the skeleton. Along this orthogonal line, the pixel intensities are extracted and recorded. Similar to [11], the width of the profile is increased by also adding intensity from neighboring parallel lines. An average of several such intensity profiles represents a wider profile, calculated at the given pixel. Intensity profiles are extracted both for the structural marker (Zo-1) and the functional marker (Bsep).

**Figure 3:**
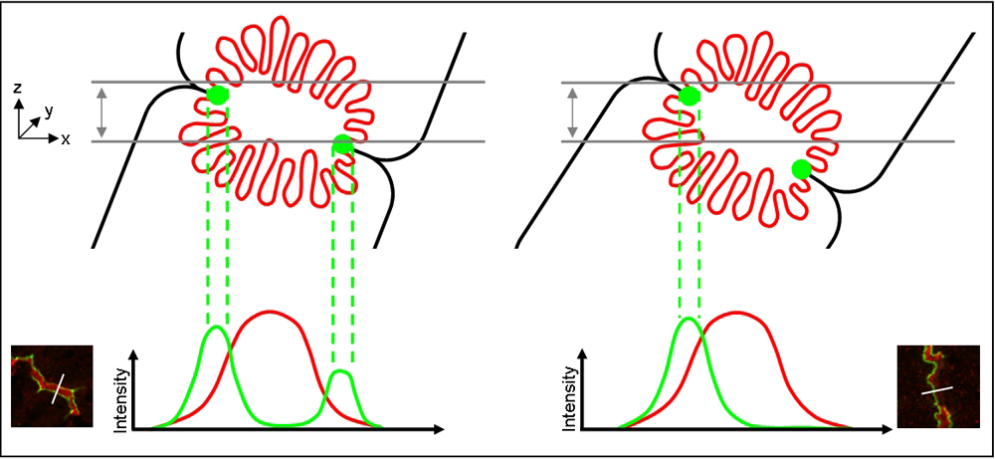
**Correlation of focal plane orientation and imaging of subcellular structures**. Due to optical sectioning, images from confocal microscopes represent thin slices of a specimen with an axial resolution of ≈ 500 nm (indicated in gray). However, in many cases the image plane might not match the orientation of the canaliculi. Depending on the orientation of the hepatocyte, the image plane might not cut both tight junctions, which delimitate the canaliculus, in the same plane. Consequently, the corresponding Zo-1 intensity profile does not exhibit two symmetrical peaks. The automated profile selection algorithm eliminates Zo-1 intensity profiles with relative peak intensity discrepancy of more than 15%. The illustration gives two examples with representative microscopic images.

Only a subset of the extracted intensity profiles clearly represent the translocation phenomenon. For example, in confocal imaging, profiles are not representative if the focal image plane does not cut the canaliculus in the principal axis (Figure 3). Humans select membrane fragments that are symmetrical, contrasty and clean. In order to implement such a strategy, Zo-1 intensity profiles are selected according to the four conditions described below.

Firstly, membrane segments non-parallel to the image plane are filtered out. Profiles extracted there exhibit Zo-1 intensity peaks of unequal height, indicating that the image plane does not match the orientation of the canaliculus. Therefore, the first condition restricts the height difference between the two local maxima. The second condition constrains the distance between the two peaks, as the variation range of canalicular widths is known. The third condition eliminates intensity profiles where the local minimum between the two peaks is not low enough (low contrast). A small intensity difference between Zo-1 peaks and valley might be caused by a damaged tissue region. It may also indicate that the focal plane is above or below the tight junctions, resulting in a low Zo-1 intensity. High quality Zo-1 profiles have two prominent peaks separated by a low local minimum, and have flat tails on the sides. Noisy profiles, in contrast, show further local maxima, and are eliminated by the fourth selection criterion. Only if all four conditions are fulfilled, a particular Zo-1 intensity profile is considered to be valid. The respective Bsep intensity profile is extracted along the same orthogonal line and is passed to further analysis.

Bsep profiles can be normalized for a quantitative analysis in several ways. Firstly, the lateral coordinate systems of profiles can be centered, compensating for skeleton lines not centered in the canaliculus. This might be caused by the skeletonization algorithm, as it uses only approximate foreground regions whose borders are not necessarily symmetric with the Zo-1 maxima. Secondly, protein distribution profiles can be normalized to a standard distance between peaks. However, in the samples we have analyzed so far, variations of the canalicular width were sufficiently small to not require such a scaling. And lastly, absolute intensity values of the profiles can be scaled to a defined range (e.g. [0, 1]). Our zonal descriptors presented in the next section, however, obviate the need for such a normalization.

The number of the extracted intensity profiles after the automated selection is still much larger than in the manual method. Therefore, we rank their quality and use only the best profiles for the statistical evaluation. The parameters of the selection process are used for the ranking procedure. The first selection criterion (difference of peak intensities) is used for the ranking *r1*. The second parameter (peak - valley contrast) leads to the ranking *r2*, defined as the ratio of the central valley height to the peaks height. The smaller the values of the criteria, the higher the ranks assigned. Later, an unweighted combination of *r1 *and *r2 *is calculated as the final ranking, according to which 20 or 30 profiles are taken for further analysis.

#### Toponomic characterization

**Figure 4:**
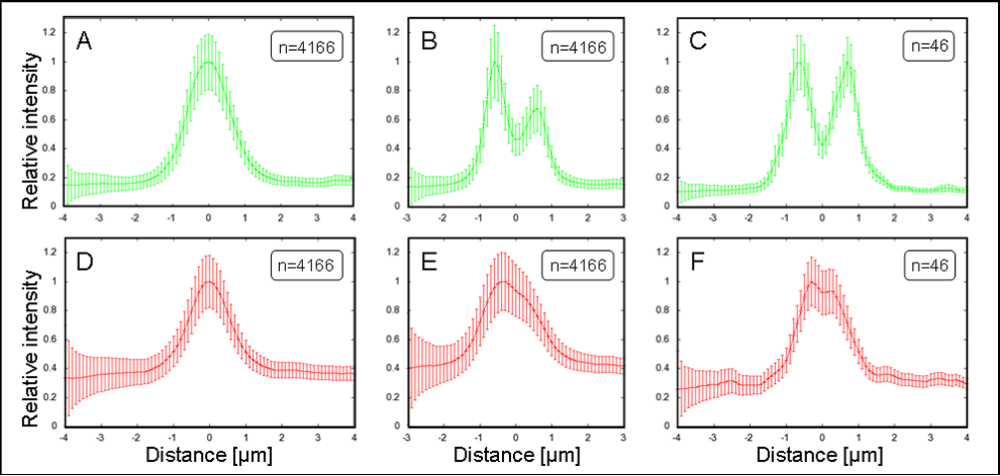
**Averaged fluorescence intensity profiles with standard deviations**. Data is shown for the first image of dataset K1 (Bsep, functional marker protein - red; Zo-1, structural marker protein - green). A. All Zo-1 profiles (n = 4166). B. All centered Zo-1 profiles (n = 4166). C. Accepted Zo-1 profiles after the selection procedure (n = 46). D. All Bsep profiles. E. All centered Bsep profiles. F. Accepted Bsep profiles.

Average Bsep and Zo-1 intensity profiles can be evaluated visually for each image. Due to the selection process, these averages already reveal the biological structure well (tight junctions correspond to the two symmetrical peaks in Zo-1 distribution), as Figure 4C and 4F show. Nevertheless, for a toponomic analysis, quantitative descriptors have to be established that reliably distinguish different protein distributions according to the underlying biological condition. For the chosen biological model, we assume a lateral translocation of the transport protein Bsep along the profile axis (center to periphery, or membrane to cytoplasm) with unknown extremes of regulation. We are therefore looking for a numerical descriptor that varies with the transporter distribution across the canalicular membrane and maximally distinguishes positive and negative controls.

**Figure 5:**
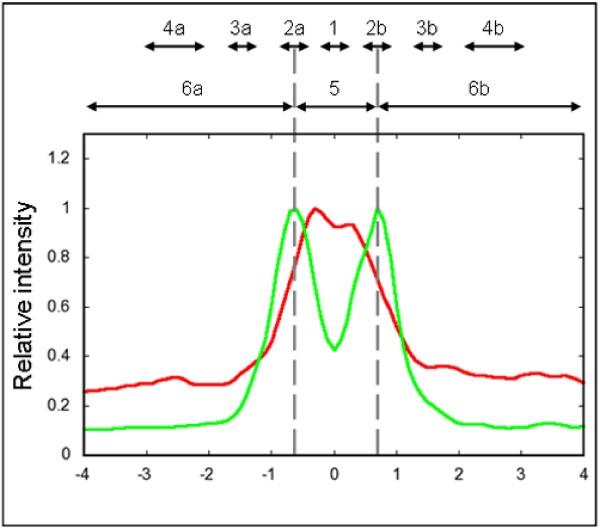
**Zone model for intensity profiles**. Zones with fixed length are calculated for the accepted Zo-1 profiles. Firstly, two peaks are identified, and the center of the profile is found. Zone 1 has a length of 5 pixels (0.5 *μ*m), and is centered between the peaks. Its length was chosen according to the empirical determination of canalicular width (0.8 - 2.5 *μ*m). The integral intensity of zone 1 reveals the amount of Bsep in the center of the canaliculus. Zones 2a and 2b (5 pixels each) are centered on the peaks and indicate protein concentration in the canalicular membrane. To analyze the internalization of transport proteins, intracellular zones were defined. Zones 3a and 3b (5 pixels each) describe intracellular fluorescence intensities close to the canalicular membrane. Zones 4a and 4b are situated closer to the centers of hepatocytes and measure 10 pixels each. Zone 5 covers the fluorescence intensities in the interior of the canaliculus between the peaks, while zone 6a and 6b combine the intracellular intensities of the profile from the maximum values of the peaks to the ends.

Such descriptors can be applied to an average profile as well as to all individual profiles. We have evaluated a number of different descriptors to identify those achieving the highest discrimination rate. In previous studies, protein distribution was described by the statistical variance of the Bsep or Mrp2 intensity profiles [6,7,10,11,13] and no profile subregions were distinguished. However, different zones can be clearly defined in the profiles. The part of the profile between the Zo-1 peaks (tight junctions) is considered to be the interior of the canaliculus, while the parts outside the tight junctions are considered to be cytoplasm. Restricting the analysis to particular zones (subregions) might improve statistical significance of a descriptor. Zones are identified based on the Zo-1 profiles and are applied to the respective Bsep profiles. Figure 5 illustrates our zone model.

Because of the fixed-length zones, integrals of the intensities can be compared as absolute numbers within one image. However, immunofluorescence methods do not allow absolute quantity measurements between images. Therefore, relative descriptors of the protein distribution are preferred, such as ratios of intensity integrals. In the following, a number of different descriptors will be evaluated (see Table 1). Two of them are presented as examples:

**Table 1:** Descriptor formulas developed for the evaluation of the protein translocation

Descriptor	Formula
X	sum1/sum2

Y	sum1/sum3

Z	sum1/sum4

A	sum1/(sum2 + sum3)

B	sum1/(sum3 + sum4)

C	sum2/sum3

D	sum2/sum4

E	sum2/(sum3 + sum4)

F	sum5/(sum5 + sum6)

(1) $$D=\frac{sum\left(Zone2a+Zone2b\right)}{sum\left(Zone4a+Zone4b\right)},$$

(2) $$F=\frac{sum\left(Zone5\right)}{sum\left(Zone5\right)+sum\left(Zone6a+Zone6b\right)},$$

Descriptor D is calculated as a ratio of Bsep fluorescence intensities at the peaks of the Zo-1 profile relative to the intensities of Bsep in the cytosol far from the canalicular membranes. Compared to control, the values of this descriptor are expected to decrease under cholestatic conditions. Bsep internalization affects *sum(Zone2a + Zone2b) *by broadening of the Bsep intensity profile. It also leads to an elevated Bsep fluorescence intensity in the cytoplasm due to immunoreactive vesicles, increasing *sum(Zone4a + Zone4b)*. As the lengths of zones are kept constant for all profiles, values of the descriptor can be easily compared even between the images. Descriptor F (internalization degree) represents a ratio of the Bsep intensity inside the canaliculus relative to the total Bsep intensity in the profile.

### Validation

Two representative image datasets of rat livers were prepared by the University Clinic Düsseldorf and will be referred to as K1 (control samples) and T1 (liver samples with induced experimental cholestasis), respectively. K1 and T1, each contain 10 images of different regions of the respective samples. It is known that Bsep is retrieved from the canalicular membrane under cholestatic conditions [31]. The expected result of the automated translocation analysis is to detect less Bsep in the canalicular membrane and an increased amount of Bsep in intracellular vesicles in T1 relative to K1.

The automated quantification workflow was applied to the datasets K1 and T1. Image processing followed by the automated profile extraction resulted in approximately 4000 profiles per image. Profile selection reduced this number to roughly 150 per image. Figure 4 illustrates improvements of the average plots of all profiles (n = 4166) after the centering (n = 4166) and after the profile selection (n = 46) for the first image of K1.

Then, 10 profiles per image were extracted manually by another expert. Descriptor values and statistical variances were computed for all automatically extracted and all manually obtained Bsep profiles. Median and standard deviation values indicate a strong correspondence between the automatic and manual results (see Table 2). Descriptor histograms show that both methods lead to almost equal distributions of variables.

**Table 2:** Descriptor values for the dataset K1.

Descriptor	Automated		Manual	
	
	Median	Stdv	Median	Stdv
C	1.93	0.624	1.97	0.639

D	1.31	0.494	1.15	0.380

Internalization Degree	0.558	0.185	0.514	0.149

Variance	0.056	0.017	0.056	0.015

Comparison of manual and automated evaluation

As distributions of the descriptor variables are not known by default, a generally applicable statistical test has to be chosen. The Wilcoxon rank sum test does not make any assumptions on the variables' distribution [11]. Furthermore, the number of extracted profiles is not known in advance and may vary between the datasets. Hence, the unpaired Wilcoxon rank sum test is chosen. It describes whether distributions of the descriptor variables differ significantly or not. Two samples are considered to be significantly distinct if the p-value is smaller than 0.05.

Wilcoxon rank sum tests were performed on the calculated descriptor values for the comparison of the datasets K1 and T1. The null hypothesis assumed that no internalization of Bsep took place in T1 (experimental cholestasis) relative to K1 (control conditions). As the Wilcoxon rank sum test is sensitive to the number of data points, we evaluated the automated and the manual methods with equal sample sizes (100 vs. 100 profiles). We randomly selected 100 profiles from those automatically extracted and selected for each dataset. The corresponding descriptor values were used for the statistical evaluation. These operations were repeated 100 times, and the median p-values are reported in Table 3.

**Table 3:** Comparison of the datasets K1 (control) and T1 (induced cholestasis)

Descriptor	P-value	
	
	Manual	Automated
X	1.9 e-1	2.0 e-1

Y	4.8 e-4	8.3 e-6

Z	8.9 e-5	1.9 e-7

A	2.9 e-1	6.1 e-2

B	8.5 e-5	6.8 e-7

C	1.3 e-6	4.5 e-13

D	9.3 e-8	8.7 e-14

E	4.0 e-8	7.6 e-15

F	6.5 e-1	4.7 e-4

Variance	6.8 e-10	2.6 e-8

P-values obtained in the Wilcoxon rank sum tests performed on descriptor values. For the automated method, median values from 100 random sampling evaluations are reported.

**Figure 6:**
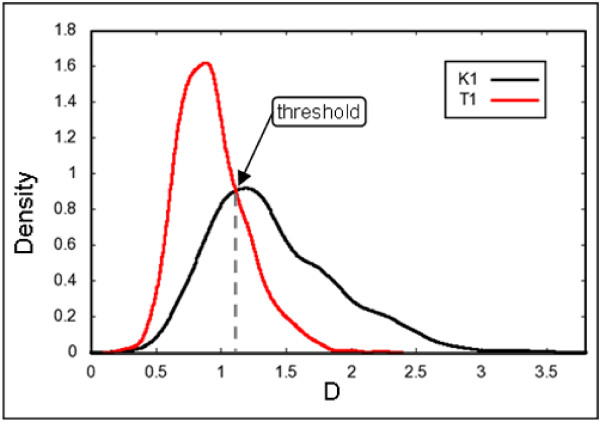
**Density plots of the values of descriptor D**. The crossing point of the two distributions (for the dataset T1 - red, for the dataset K1 - black) is defined as a threshold for discrimination of the datasets. A trivial classifier can be trained and assessed by precision and recall values. Distributions are significantly distinct, but overlap is still large.

Additionally, a simple classifier was trained to evaluate whether individual descriptors are suitable for diagnostic discrimination between different biological conditions (Figure 6). Such an evaluation was performed for the statistical variance and the descriptor D, which exhibited one of the lowest p-values among the newly developed descriptors. A crossing point of the variable's density plots (from the positive and the negative control) was set as a threshold for the discrimination. After the prediction, the classifier was assessed by its precision and recall values [32] (see Table 4).

**Table 4:** Simple classifier performance for the comparison of the datasets K1 and T1

	Precision, %	Recall, %	F-score, %
Variance	71.02	65.80	68.31

D	75.80	84.76	80.03

Automated and manual evaluation were reproduced in three independent cholestatic liver preparations and the respective control animals (K2, K3 (controls) and T2, T3 (induced cholestasis)). Bsep internalization was confirmed in all test samples with comparable p-values. As expected, the difference between the control datasets (K2 and K3) was small and reported p-values were high (see Table 5). In contrast, comparison of K2 and T2 (data not shown) and K3 and T3 (see Table 6) showed a highly significant difference in Bsep distributions due to the internalization under experimentally induced cholestasis. In both cases, the results were improved by using top-ranked selected profiles. Mean values of the descriptors were compared for these data (see Table 7).

**Table 5:** Comparison of the control datasets K2 and K3

Descriptor	P-values		
	
	Not ranked	30 Top-ranked	20 Top-ranked
C	1.2 e-1	5.8 e-1	5.6 e-1

D	5.1 e-3	2.6 e-1	2.4 e-1

Variance	9.0 e-2	3.4 e-1	5.7 e-1

P-values obtained in the Wilcoxon rank sum tests performed on descriptor values from all accepted or the top-ranked accepted profiles.

**Table 6:** Comparison of the datasets K3 (control) and T3 (induced cholestasis)

Descriptor	P-values		
	
	Not ranked	30 Top-ranked	20 Top-ranked
C	1.3 e-11	2.5 e-13	8.0 e-14

D	3.8 e-6	2.8 e-8	7.7 e-9

Variance	2.9 e-4	1.5 e-7	5.0 e-7

P-values obtained in the Wilcoxon rank sum tests performed on descriptor values from all accepted and the top-ranked accepted profiles.

**Table 7:** Descriptor values for datasets K2, K3 and T3 (using 20 top-ranked accepted profiles)

Descriptor	K2		K3		T3	
	
	*μ*	*σ*	*μ*	*σ*	*μ*	*σ*
C	1.878	0.560	1.909	0.594	1.303	0.360

D	1.354	0.453	1.293	0.478	0.947	0.354

Variance	0.055	0.014	0.057	0.016	0.045	0.014

## Discussion

Rapid changes in transporter localization by endo- and exocytosis of transporter-bearing vesicles from and into the respective cell membrane have been identified as an important regulatory mechanism for cells to adapt to varying conditions. Previous studies of the membrane transport protein distribution by manual analysis of fluorescence microscopy images were time consuming and error-prone [6,7,10,11,13,14]. We developed an automated quantification of the membrane protein distribution for fast and objective evaluation of experimental datasets.

### Image processing

Initial image processing with the Zeta software has demonstrated to be fast (max. 1 minute) and robust for images of different sources. Foreground detection results were in all cases acceptable, as no high contrast foreground objects were misclassified. Varying grades of background fluorescence, presence of further markers and staining artefacts did not disturb the stable performance. This illustrates the advantage of machine learning of model parameters over simple thresholding.

Variation in the performance of the foreground-background detection has only limited influence on final results. Firstly, good membrane fragments might be missed (false negatives) due to bad training examples given or low contrast to the background. Secondly, background regions might be classified as foreground (false positives) if the training examples have not been specific enough. In this case, the profile selection procedure will filter out bad profiles (those without two significant peaks in Zo-1 channel). And lastly, membrane fragments can be segmented with wrong borders, shifting a skeleton line from its optimal position. Consequently, the respective intensity profiles will also be axially shifted relative to the center of the membrane fragment. However, profile selection will ensure that only acceptable intensity profiles are used for the statistical analysis. Therefore, weak performance of the object detection step may lead to a reduction of the number of profiles, but will not influence the further profile evaluation.

Image processing was optimized on model images with 512 × 512 pixel resolution and a pixel size of approximately 300 nm. Thresholds for the deletion of membrane segments were manually selected so that all big high contrast canalicular membranes with two parallel Zo-1 intensity lines were kept. Several skeletonization techniques were tested on membrane fragments, and the simplest one was selected, as the other techniques did not affect the quality of the selected profiles. Generally, our method yields comparable results even with altered threshold configurations. The robustness of the method was tested on images from other sources and with different pixel sizes.

### Profile optimization

Microscopic images of the datasets K1 and T1 were recorded with 1512 × 1512 pixel resolution and a pixel size of 100 nm. Approximately 4000 individual profiles per image were automatically extracted. Initial average profiles of the structural marker (Zo-1) generally did not reveal the structure of tight junctions, due to a high level of variation of individual profiles. After the profile selection, average intensity profiles were comparable to those manually extracted. As in the manual profile extraction, only long, straight and symmetrical membrane fragments were considered. We could show that the introduction of the criteria for profile selection could substitute manual profile extraction based on experience and biological knowledge (see Table 2, Table 3).

Both selection and peak-to-peak centering of the individual profiles led to a significant improvement of the average profiles. A Gaussian-like distribution of Zo-1 (Figure 4A, all points) did not comply with the expected structure of tight junctions. It became visible in the average profiles of the centered or selected points (Figure 4B and 4C, respectively). However, only the average over the selected profiles (Figure 4C) showed two symmetrical peaks and significantly reduced standard deviations.

There were approximately 150 profiles per image remaining after the selection procedure, compared to 10 profiles, which were manually extracted by biologists. Consequently, the automated workflow does not only speed up the process and clean the data, but also increases the number of data points obtained for the analysis.

The suggested Zo-1 profile selection procedure was tested for robustness and stability. Two of the four criteria were varied, and numbers of accepted profiles were reported. The first threshold constrains the allowed height difference of the two Zo-1 peaks. The second threshold limits the central valley height between these peaks. Table 8 shows the numbers of accepted profiles from the total of 2871 individual profiles extracted from the first image of the dataset K1. The rejection percentage was found to be slightly more sensitive to the first threshold. However, no extreme dependencies on the threshold values were recorded.

**Table 8:** Rejection percentage

		Difference of peak height <			
		
		5%	15%	25%	35%
Valley height <	10%	32	107	151	194

	20%	45	166	230	294

	30%	56	179	264	333

	40%	58	187	273	351

Numbers of accepted profiles (out of 2871) at different thresholds for the selection criteria.

### Descriptors for profiles and statistical tests

Quantification of the transport protein distribution was significantly improved by the introduction of the zone model. Earlier, only statistical variances of protein intensity profiles were used [6,7,10,11,13]. Subdivision of the intensity profiles into biologically meaningful zones improved the quantitative separation of the toponomic states. Wilcoxon rank sum tests demonstrated that the difference in protein distribution between images from cholestatic and control animals is more significant for the new descriptors (see Table 3, automated). However, the new descriptors did not outperform the statistical variance when evaluated at the manually extracted profiles (see Table 3, manual).

P-values obtained in the Wilcoxon rank sum tests on the new descriptors differed significantly among each other (from e-1 to e-15, see Table 3). One factor seems to be the selection of zones employed in the respective formula. For example, those descriptors including intensities in zone 1 (center of the canaliculus), e.g. X and A, performed worse. In contrast, descriptors, which did not include zone 1, namely C, D and E, led to the most significant results, which may have physical reasons. Confocal fluorescence microscopy is an optical sectioning technique, which acquires images of thin slices (≈ 500 nm) of a thick specimen [33]. Therefore, vesicle movement perpendicular to the image plane will not change the intensity, while lateral vesicle movement within the image plane can be assessed by intensity distribution profiles. If there is a low Bsep signal in the zone 1, it could be caused by two different situations, which are indistinguishable for a confocal microscope. Either the focal plane contains only a canalicular lumen, or the canalicular microvilli membrane in the focal plane has a reduced Bsep level due to the out-of-plane transporter vesicles. Thus, intensity in the zone 1 is not directly related to membrane content. Zone 1 can apparently be neglected in intensity profile analysis, which illustrates the advantage of the introduction of profile zones.

Results of the Wilcoxon rank sum tests suggested a significant difference between images from the datasets K1 and T1. Still, further evaluations showed generally significant overlaps in the density plots of the descriptor values. An example of such a density plot is shown in Figure 6. Training of a simple classifier allowed better estimation of the descriptors' differentiation facilities. A model trained on the values of the descriptor D exhibited an F-score [32] of approximately 80%. In comparison, a classifier trained on the statistical variance reached an F-score of only 68%.

The internalization degree (descriptor F) represents an intuitively understandable measure. In control animals, image analysis showed that approximately 56% of the total fluorescence intensity of the Bsep transporter was localized between the two Zo-1 intensity maxima (canalicular membrane). Experimental cholestasis led to a reduction of the descriptor F value to 47% caused by the lateral Bsep translocation from the canalicular membrane towards hepatocytes' lumen. Despite being physically understandable, the internalization degree did not yield the best p-values in Wilcoxon rank sum tests and was not optimal to quantify transporter internalization.

Ranking of the selected profiles has proven to have an impact on the evaluation of the datasets. A stronger internalization effect was detected for K3 - T3 datasets when using the top-ranked in comparison to all selected profiles (see Table 6). Furthermore, two control datasets (K2 and K3) were found to be more similar based on the top-ranked profiles (see Table 5). Consequently, the ranking procedure made statistical tests more sensitive.

Statistical evaluation of the datasets of the same biological condition (e.g. comparison of K2 and K3 (see Table 5), or T2 and T3 (data not shown)) revealed that the difference between their Bsep distribution is small. It can be explained by biological variability and limitations of standardization in sample preparation. As expected, the difference between cholestatic and control datasets was much larger with very low p-values (compare Table 5 and Table 6).

### Automated vs. manual method

The significance of statistical tests was much lower for the manual method in comparison to the tests on all of the automatically extracted and selected profiles, due to the smaller number of profiles (data not shown). However, when using equal sample sizes, a very good correlation of p-values can be noted between the manual and the automated methods (see Table 3). Descriptors X and A were the least eligible, while D and E showed the best performance.

Initially, automatically extracted profiles contained noise and were measured partly at the membrane fragments unsuitable for the analysis. The introduction of the selection procedure improved the quality of the average intensity profiles, as shown by the reduced standard deviations (see Figure 4). Statistical tests on the automatically extracted and selected profiles confirmed the expected difference between the positive and negative controls, and correlated well with the results from the manually obtained profiles.

Furthermore, the automated workflow processed a dataset in approximately 30 minutes and performed statistical tests between the datasets in about 15 minutes, while a human would need several hours for such evaluations.

The software would be freely available on request to anyone wishing to use it for non-commercial scientific purposes.

## Conclusions

We have developed an automated method to analyze transport protein toponomics and compared it to the known manual quantification. The automated intensity profile extraction is faster and acquires a larger number of data points than the manual method. Furthermore, the criteria suggested for the profile selection are fully reproducible. Evaluation of the automatically and manually extracted data correlated well. Several points contribute to this result. Firstly, automated profile selection is comparable to the manual profile extraction based on the biological knowledge. Secondly, introduction of the zone model improved the results by identification of regions, which are particularly meaningful regarding the internalization of membrane proteins. The suggested descriptors characterize the datasets better than statistical variances of complete intensity profiles. The internalization degree descriptor has a clear physical meaning and illustrates the protein retrieval from the membrane to decrease negative effects of bile acid secretion under cholestatic conditions [31]. However, it did not perform best among the newly developed descriptors.

The new method was tested with various configurations. Even outside the optimal settings, robust results are produced. The same experiments were performed on 5 further datasets, including samples from a different experimental setup (internalization of Bsep under hyperosmolar conditions). In all these cases a good correlation with manually obtained results was shown. Consequently, the developed translocation analysis has proven to be robust and to exhibit stable performance on various datasets.

## Methods

Rat liver tissue is used to develop an automated quantification of the membrane protein translocation on the example of the cholestatic liver diseases. Ligature of the common bile duct is used as a model for experimental cholestasis. Extension of the evaluation method to human liver samples is planned.

### Rat bile duct ligature

The experiments were approved by the responsible local authorities. Following general anesthesia, male Sprague Dawley rats underwent double ligature of the proximal common bile duct or sham operation (control animals) as described previously [34]. Livers were removed 7 days after a bile duct ligation or a sham operation.

### Rat liver perfusion

Livers of male Wistar rats were perfused *in situ *as described previously [6,7]. After a perfusion period of 20 minutes with normosmotic (305 mosmol/L) perfusion buffer, a liver lobe was ligated, excised, and frozen in isopentane precooled in liquid nitrogen (t = 0 minutes). Liver perfusion was continued for additional 30-minute periods with perfusion buffers of a desired osmolarity (305 mosmol/L or 405 mosmol/L) until a second (t = 30 minutes) liver lobe was removed.

### Cryosectioning and immunostaining of rat liver

Sample preparation and immunostaining were performed according to a standard operating procedure to assure reproducibility. The tissue samples were cut in 5 *μ*m sections with a Leica Cryotom CM1950 (Leica, Bensheim, Germany) and fixed with methanol for 10 minutes at -20°C. Washing steps were performed in a washing station for slides (Advalytix AdvaWash, Implen, Germany) for 15 minutes. Sections were sequentially incubated in a microarray hybridization station (Advalytix Slide Booster SB450, Implen GmbH, Germany) with a combination of the primary antibodies (rabbit anti-Bsep, K12, 1:30 and mouse anti-Zo-1, 1:750) for 2 hours at 28°C and a combination of the secondary antibodies (Alexa Fluor 488-conjugated goat anti-mouse, 1:500, green; Alexa Fluor 546-conjugated goat anti-rabbit, 1:500, red) for 30 minutes at 37°C.

### Image acquisition

Immunostained rat liver tissue samples were analyzed using a LSM 510 confocal laser scanning system (Zeiss, Jena, Germany) with a 63 × Plan-Apochromat objective (NA 1.4). The excitation wavelength was 488 nm for Alexa Fluor 488 and 543 nm for Alexa Fluor 546. Emission was detected by a 505 - 530 nm (green) and a 560 - 615 nm bandpass filter (red). Image acquisition was adjusted to a final pixel size of 100 nm. For each cryosection, images from 10 different regions were taken in a randomized fashion and used for the analysis.

### Manual determination of regions of interest and measurement of fluorescence intensity profiles

Cryosections of rat liver were stained for the functional marker Bsep and the structural marker Zo-1. The tight junction protein complex forms the border between canalicular and sinusoidal membranes. The areas for the analysis were chosen by assessing the apparent integrity of the canaliculi. Regions of interest were found where the immunostaining of Zo-1 delineating the bile canaliculi were undisrupted and parallel lines. Canalicular segments were expelled when they were bent, small or non uniform. Figure 1A shows an example of a membrane fragment suitable for analysis. Bsep and Zo-1 distributions were analyzed by extracting intensity profiles of the red and green fluorescence perpendicular to the canaliculi using the Profilizer software (developed by Martin Becker). With this software users can view images and interactively select the position of intensity profiles. A user has to click two points symmetrically to a membrane fragment in a microscopic image, and the Profilizer will draw a line between them (an intensity profile direction). It will also cut the line to 4 *μ*m on both sides of the membrane and output diagrams of pixel intensities for each of the channels (the intensity profiles themselves). These manually extracted intensity profiles are exported for further evaluation.

The length of extracted intensity profiles was 8 *μ*m (Figure 1A), corresponding to 81 pixels with a pixel size of 100 nm. Intensity profiles were accepted according to the appearance of Zo-1 fluorescence. Acceptable intensity profiles had two maxima of similar size and a minimum between these peaks close to baseline level. An empirical image analysis showed that canalicular diameter varies in the range of 0.8 - 2.5 *μ*m. Therefore the profiles were excluded if the distance between the two maximal intensities was < 10 or > 25 pixels. The immunostaining of Bsep was disregarded for profile selection, thus data acquisition was performed in a blinded fashion. Ten intensity profiles were selected per image. This manual analysis is subjective, as an expert makes the decision where to extract the profiles based on their experience. Moreover, the method is time consuming and error-prone.

### Parameters for the automated image processing

A square window of 15 pixels was used for the texture feature extraction. Morphological operations were performed with a 3 × 3 structural element. The first thresholding eliminated objects with an area of less than 125 pixels. The following threshold was set to 250 pixels and was greater than the previous one, because some gaps were possibly closed and bigger fragments were formed.

The applied skeletonization was based on the Euler number for polygon networks and polyhedra:

(3) E=e-k+f,

where *e *is the number of corners, *k *defines the number of edges, and *f *is the number of faces [30]. An image was scanned with 3 × 3 pixel window and an Euler characteristic was calculated. Only those pixels that had more than one foreground neighbor pixel were considered for the deletion, in order to avoid shortening lines from their ends. A pixel with at least two foreground neighbors was then deleted if this operation did not change the Euler number of the window, which indicated the number of connected components. This operation was performed until no further pixels could be eliminated without changing the Euler characteristics. Skeleton fragments left after pruning and deletion of branching points were cleaned from objects smaller than 7 pixels.

All thresholds mentioned above were identified empirically for microscopic images with a resolution of 512 × 512 pixels (a pixel size of ca. 300 nm) and were evaluated for the maximal robustness of the method. For other resolutions, the values can be scaled accordingly.

### Intensity profile extraction, evaluation and normalization

The length of the calculated profiles should cover approximately 8 *μ*m, which translates to 81 pixels (nearest odd value) for our images. To determine whether the length of 81 pixels is sufficient, profiles of a length up to 501 pixels (50 *μ*m) were calculated and compared. Rat hepatocytes have a diameter of 20 - 30 *μ*m [35], so that a profile of 501 pixels covers approximately the fluorescence intensity distribution of one hepatocyte on each side of the canaliculus. It was found that the intensity of Bsep stayed nearly constant for distances of more than 35 pixels from the center point. Consequently, a total profile length of 81 pixels is sufficient.

The width of the profiles was varied from 0.1 to 1.1 *μ*m (which corresponded to [1 - 11 pixels]). A width of 0.3 μm was found to be the most robust and least biased towards good profiles.

Formulas for the profile selection criteria are presented below:

(4) $$\frac{maxValue\text{1}-maxValue\text{2}}{maxValue\text{2}}<0.15,$$

(5) $$7<∥\left(positionMax1-positionMax2\right)∥<26,$$

(6) $$\frac{minValue}{maxValue2}<0.2,$$

(7) $$\frac{P_{60}\left(localmaxima\right)}{maxValue2}<0.4,$$

where *maxValue1 *is the absolute maximum of the Zo-1 intensity profile, *maxValue2 *is a height of the second local maximum, and *minValue *defines the intensity of the local minimum between the considered two local maxima. Positions of the peaks on the profile are *positionMax1 *and *positionMax2*, respectively. *P*_60 _*(local maxima) *is the 60*^th ^*percentile of the local maxima values.

The relative difference between the peaks (4) should not exceed an empirically identified threshold of 15%. The second condition (5) constrains the distance between the two peaks, as the diameter of the canaliculi varies in the range 0.8 - 2.5 *μ*m. The third criterion (6) filters out profiles with a valley not deep enough. The last requirement (7) eliminates noisy profiles.

For centering of the lateral coordinate systems of intensity profiles, firstly, a model profile is required that defines necessary shifts. For this purpose an average Zo-1 profile over all accepted profiles is calculated. Because of the strict selection of profiles with two clear peaks and the moderate amount of decentering in such regions, the average of all accepted profiles will still have two clear peaks, which define the average location and width of canaliculi. Initially, the calculated model profile is centered itself. Two peaks are detected and the midpoint is set as the null point of the model profile. The profile is cut to the length of 61 pixels to allow cross-correlation (dot product) between Zo-1 profile and the model profile to be calculated in 20 different positions (+/- 10 pixels shift). Then, the Zo-1 profile is overlaid with the shifted model profile. The maximum among these dot products determines the optimal shift. Both, individual Zo-1 and Bsep profiles, are shifted respectively and cut to the length of 61 pixels.

## Authors' contributions

OD and SB have implemented the automated image processing and intensity profile extraction. OD has developed the selection criteria, and scale-free descriptors in collaboration with SM. SM and RK have performed the biological experiments with rats and acquired the microscopy data. MB has manually evaluated the microscopy data according to a known method. OD has performed the automated analysis of the data and statistical tests. RK, DH and TB have supervised the work and contributed to the text. All authors read and approved the final manuscript.
